# Epicutaneous Immunotherapy for Aeroallergen and Food Allergy

**DOI:** 10.1007/s40521-013-0003-8

**Published:** 2013-12-17

**Authors:** Gabriela Senti, Seraina von Moos, Thomas M. Kündig

**Affiliations:** 1 Clinical Trials Center, University Hospital Zürich, Zürich, Switzerland; 2 Department of Internal Medicine, University Hospital Zürich, Zürich, Switzerland; 3 Department of Dermatology, University Hospital Zürich, Gloriatrasse 31, 8091 Zürich, Switzerland

**Keywords:** Epicutaneous allergen-specific immunotherapy, Transcutaneous allergen-specific immunotherapy, Respiratory allergy, Food allergy

## Abstract

IgE-mediated allergies today affect up to 30 % of the population in industrialized countries. Allergen immunotherapy is the only disease-modifying treatment option with a long-term effect. However, very few patients (<5 %) choose immunotherapy, due to the long treatment duration (between 3–5 years) and possible local and systemic allergic side effects of the allergen administrations. The latter occur when an allergen accidentally reaches the blood circulation. Therefore, the ideal application route for allergen immunotherapy should be characterized by two hallmarks: firstly, by a high number of potent antigen-presenting cells, which enhance efficacy and thus shorten treatment duration. Secondly, the allergen administration site is ideally non-vascularized, so that inadvertent systemic distribution of the allergen and consequent systemic allergic side effects are minimized. The epidermis contains high numbers of potent antigen-presenting Langerhans cells and, as an epithelium, is non-vascularized. Therefore, the epidermis represents an interesting administration route. Historical evidence for the clinical efficacy of epicutaneous allergy immunotherapy (EPIT) has now been strengthened by a number of recent double-blinded placebo-controlled clinical trials performed by independent groups. We review the immunological rationale, history and clinical experience with epicutaneous allergy immunotherapy.

## Introduction

With a current prevalence of up to 30 % in industrialized countries, IgE-mediated allergies have become an important socioeconomic burden. Symptomatic treatment with antihistamines and corticosteroids can efficiently ameliorate IgE-mediated symptoms [1], but does not stop progression of the underlying allergy. The only disease-modifying treatment is allergen immunotherapy (AIT) [1, 2]. Subcutaneous allergen-specific immunotherapy (SCIT) was introduced more than a century ago by Leonard Noon [3], but has two major disadvantages: it is time-consuming, as it requires 30–70 visits to a medical practice, and subcutaneous allergen injections are associated with local and systemic allergic side effects [4–6]. These two main drawbacks may potentially be resolved by the following strategies.

First, to reduce the number of injections, immunogenicity of the allergen administration must be enhanced. This may theoretically be achieved (1) by increasing the allergen dose. While a dose effect in AIT is evident [7, 8], allergic side effects prohibit significant dose increases. Use of hypoallergenic allergens by chemical modification to allergoids [9], by recombinant modification [10] or by using non–IgE-binding peptides [11, 12, 13••], may permit increased allergen doses, but could reduce allergen immunogenicity. A reduction of injection numbers may also be achieved (2) by replacing the classical adjuvant alum, which is considered to actually favour a Th2 response, with a Th1-promoting adjuvant such as the Toll-like receptor (TLR) ligands CpG [14•] or MPLA [15–17]. The number of injections may also be reduced (3) by allergen delivery via a route characterized by a high density of antigen-presenting cells. These are present at highest density in secondary lymphatic organs such as lymph nodes, and indeed, when administered intralymphatically, the number of allergen injections could be reduced to only three [18•, 19•, 20].

Second, to improve the safety of AIT, inadvertent allergen delivery to the blood vasculature must be avoided. Ideally, the allergen should be delivered to a non-vascularized tissue. In certain ways, sublingual allergy immunotherapy (SLIT) fulfils this criterion, as the allergen is delivered to the oral mucosa, which is covered by a multi-layered epithelium. Despite diffusion of the allergen down into deeper layers containing mast cells, which are responsible for the frequently observed local oral side effects [7, 21], SLIT is considered very safe with respect to systemic allergic side effects [7, 21]. Apparently, diffusion into a vascularized layer is less risky than injection into a vascularized layer. The same should be true for epicutaneous allergy immunotherapy (EPIT), where an allergen is administered to the non-vascularized epidermis. However, as an advantage of EPIT over SLIT, keratinocytes can additionally be activated by physical irritation, e.g., abrasion or adhesive tape stripping, or by adding adjuvants [22]. Epithelial damage increases keratinocyte expression of additional molecules such as IL-1α, IL-6 and TNF-α, skewing the immune response toward a Th1-type response [23]. Such activation of keratinocytes is important for creating a pro-inflammatory environment with enhanced activation of Langerhans cells.

Hence, EPIT has the potential not only to reduce side effects by minimizing allergen penetration to the vasculature, but also to shorten treatment duration by increasing immunogenicity of the administered allergen formulation.

## Potential application fields of EPIT

Vaccination started with epicutaneous immunization, when Edward Jenner applied cow pox virus to scarified human skin [24]. Having then been forgotten for a long time, epicutaneous vaccination had its revival at the beginning of the twenty-first century, driven by the increasing interest in novel needle-free vaccination routes [25, 26]. Epicutaneous vaccination against *Escherichia coli*–induced traveller’s diarrhoea [27••] was the first step. Animal models have so far shown successful vaccination against infection with *Helicobacter pylori* [28], influenza virus [29] and diphtheria toxin [30]. The protective mechanism in all of these applications relies on induction of humoral immunity dominated by IgG1 and IgA. Studies testing epicutaneous vaccination against HIV also found induction of mucosal cytotoxic T cells together with secretion of mucosal antibodies [31]. Another field of application is cancer immunotherapy. Several groups achieved promising results with epicutaneous immunotherapy against skin cancer based on induction of potent CD8^+^ T cell responses [32, 33]. Not only has EPIT been demonstrated to induce effector T cell responses, but also suppressive T cell responses when EPIT was used to inhibit experimental allergic encephalomyelitis [34, 35].

## History of EPIT in allergy treatment

EPIT as a treatment for allergies was introduced surprisingly early. In 1917, Besredka showed that EPIT was able to induce specific antibodies [36••], and the first case study on successful epicutaneous allergy immunotherapy was reported in 1921 by Vallery-Radot, who found that allergen administration onto scarified skin reduced systemic allergic symptoms in patients allergic to horses [37•].

A decade later, when the risk of suffering a “pollen shock” during allergen immunotherapy was recognized to be a considerable danger of subcutaneously administering allergen to highly sensitized patients, a similar method—called intradermal allergen specific immunotherapy—received attention [38, 39]. Based on the observation that hay-fever patients occasionally experienced symptom amelioration after “intradermal pollen tests”, E. W. Phillips started to treat highly sensitive patients as well as patients requesting co-seasonal treatment by administration of pollen extract [39]. Strikingly, such intradermal allergy immunotherapy proved to be both safe and highly efficacious, leading to symptom relief after administration of only three doses [39]. At the same time, M. A. Ramirez treated patients allergic to grass pollen with a method he called “cuti-vaccination”, which consisted of administration of pollen extract onto scarified skin [36••]. Based on these results, it was suggested in the 1930s that the subcutaneous route might not be optimal for administration of AIT: “knowledge of the epidermis as an immunologic organ is still meager … it may be theoretically possible that a more effective desensitization may be attained by this route than by the subcutaneous one” [38].

Between 1950 and 1960 French allergologists revisited EPIT [40, 41]. Pautrizel administered the allergen extract onto slightly rubbed epidermis. Even though the reported results were excellent, a large number of applications were necessary until symptom relief was observed [40]. Blamoutier, in contrast, applied the allergen drops onto heavily scarified skin [36••, 41]: “On the proximal volar aspect of the lower arm, in a square area of 4 × 4 cm, chessboard-like horizontal and vertical scratches are made with a needle.... These scratches should be superficial and not cause bleeding” [42]. Consistently, allergic side effects were observed only rarely when allergen was applied via the skin, and if they occurred nevertheless, these reactions were always milder than under conventional SCIT [36••, 39–41]. These promising results were supported by several studies performed in the subsequent years all over Europe, from Switzerland [42, 43] to Portugal [44]. Overall, symptom relief was obtained rapidly and allowed for co-seasonal treatment. The reported treatment success rates of 80 % exceeded the success rates under conventional SCIT [42]. Despite such successful results with the French *méthode de quadrillage cutané*, reports on this promising administration route disappeared into oblivion for almost half a century.

## EPIT for aeroallegens

While there is strong scientific and historical evidence for EPIT in allergy treatment, no double-blind placebo-controlled clinical trials existed, a fact that led our group to revisit EPIT. Driven by the idea to find a patient-convenient application route of AIT in order to increase its attractiveness, and based on the good accessibility of the skin and its high density of potent immune cells, our group performed three clinical trials to test efficacy and safety of EPIT. In order to keep epithelial barrier disruption minimal, we replaced skin scarification by adhesive tape stripping [45]. Besides enhancing the penetration of allergens by removing *stratum corneum* [46], repeated tape stripping also functions as a “physical” adjuvant through activation of keratinocytes, which then secrete various pro-inflammatory cytokines (IL-1, IL-6, IL-8, TNF-α and IFN-γ) favouring maturation and emigration of DCs to the draining lymph nodes [47, 48]. Results of the first pilot trial revealed that patients treated with a total of 12 patches containing pollen extract experienced significant alleviation of hay fever symptoms compared to placebo-treated patients. In line with the “historical” study results described above, no severe systemic allergic reactions were reported. The only adverse events observed were very mild local eczematous reactions under the skin patch [45]. When looking at all 12 patch applications, mild eczema was observed in 15 out of the 21 verum patients, whereas such eczema was only seen in 5 out of the 15 placebo patients. When looking at a single patch application, eczema under the patch, with a severity score between 3 and 6 on a scale ranging from 0 to 18, was observed in roughly half of the verum patients. In order to exclude partial unblinding of the study by the occurrence of local adverse effects, we had analysed whether or not the occurrence of eczema under the patch correlated with symptom amelioration, but we could not find such a correlation. Encouraged by these results, a second phase I/IIa trial including a total of 132 grass pollen–allergic patients was initiated to find the optimal treatment dose of EPIT. Enrolled patients were treated co-seasonally with a total of six patches. We found a clear relationship between the administered allergen dose and the clinical effect, with the highest allergen dose leading to the most marked symptom improvement [49•]. Also, we found a dose-dependence of the local adverse effects where patches were applied, with pruritus being the most frequently reported adverse event, followed by eczema observed after patch removal. Interestingly, with every subsequent patch application, there was a reduction of local adverse events. After the sixth patch application, only half as many local adverse events were reported. This reduction was not explicable by local depletion of immune cells or degranulation of mast cells, as each of the six patches was applied to a different area of the arm. Therefore, the reduction of local adverse events is likely to be explained by tolerance induction. A third clinical trial investigated the immunological changes induced during EPIT and found an increase in allergen-specific IgG4 [50]. Our results have meanwhile been confirmed by an independent group that demonstrated efficacy and safety of EPIT in grass pollen–allergic children. Hay fever symptoms, as well as the use of antihistamines, were significantly reduced in the active treatment group [51•].

Also, the results of intradermal allergy immunotherapy reported by W. Philipp back in 1926 have meanwhile been indirectly confirmed in a double-blind placebo-controlled trial by Rotiroti et al. [52••]. They found that six low-dose intradermal injections with grass pollen extract, i.e., only 7 ng of Phl p 5 per injection, strongly reduced the allergen-induced cutaneous late response.

So far, there is no head-to-head comparison of EPIT to other routes of administration, except in mouse models. Using the major grass pollen allergen Phl p 5, EPIT was found in the mouse to be at least equivalent to SLIT [53]. While EPIT and SLIT induced similar IgG2a levels and also led to a similar reduction in IgE levels in sensitized mice, it was only EPIT that led could significantly reduce eosinophil counts in the bronchoalveolar lavage (BAL) in the asthma model. Also in mice, we have compared SCIT with EPIT using ovalbumin as the allergen. While EPIT without adjuvant was less immunogenic than SCIT, EPIT with an adjuvant was found to be more immunogenic, so that EPIT and SCIT appeared comparable in efficacy [22].

## EPIT for food allergens

A clinical pilot trial to test clinical efficacy and safety of EPIT using the Viaskin® EDS in children suffering from cow’s milk allergy showed a tendency toward an increased cumulative tolerance dose after a three-month treatment period, but missed statistical significance. Treatment was well tolerated, with no systemic anaphylactic reactions, but a significant increase of local eczematous skin reactions was observed [54••]. Such good safety results are crucial, especially when considering the use of EPIT as treatment option for food allergies for which conventional SCIT is impractical due to an unacceptably high rate of anaphylactic reactions [55]. To substantiate these early findings, and aiming to develop a definitive therapeutic option for food allergy patients, a phase I (NCT01170286) as well as a phase II trial (NCT01197053) have recently been initiated with the objective to test treatment efficacy of EPIT with the Viaskin® EDS in peanut allergy patients.

## Outlook

As there now exist several placebo-controlled double-blind clinical trials confirming the efficacy of EPIT in allergy treatment [45, 49•, 51•, 52••, 56], there remains little doubt that this method works in principle. Our clinical trial program has also shown that the allergen dose is an important efficacy parameter [49•]. The allergen dose used in skin patches cannot readily be increased any further. We had already used 30 μg of major grass pollen allergen Phl p 1, corresponding to 1 ml of the tenfold concentration that is used for the skin prick solution. A higher concentration would not only generate considerable cost of materials, but application to any accidentally impaired skin barrier would increase the risk for systemic allergic effects. Future research should therefore focus on enhancing penetration of the stratum corneum into the viable layers of the skin where Langerhans cells reside, and also on adjuvants suitable for epicutaneous administration.

Methods for enhancing penetration across the skin barrier include (1) hydration of the stratum corneum, which facilitates diffusion of hydrophilic molecules. Any form of occlusion, such as the patches we [45, 49•] and others [51•] have used, hydrates the skin by accumulation of sweat [54••, 57]. Also, a French group recently developed an alternative form of EPIT based on allergen delivery to the intact skin using an occlusive epidermal delivery system (Viaskin® EDS) [54••, 57, 58]. Initially developed for diagnostic purposes as an alternative system to the conventional Finn chamber used in atopy patch test [59], Viaskin® relies on the ability to deliver whole protein molecules to the skin [57, 58]. Perspiration generated under an occlusive chamber dissolves the lyophilised allergen protein loaded on the Viaskin® EDS [57, 58]. Delivered via such EDS, proteins have been demonstrated to accumulate in the stratum corneum, where they efficiently targets immune cells of the superficial skin layer [60] that rapidly migrate to the draining lymph nodes [57]. In murine studies, EPIT using the Viaskin® EDS showed efficacy equivalent to SCIT in preventing allergic airway reactions upon inhalative allergen challenge [57]. Skin penetration may also be enhanced (2) by adding so called penetration enhancers, such as salicylic acid (used by Agostinis et al. [51]) or (3) by packing the antigen into lipid based colloidal systems [61]. Last but not least, skin penetration can be enhanced (4) by microporation, either using a microneedle patch [29, 62] or a LASER [63•, 64].

While all the above methods enhance skin penetration of allergens, it remains to be seen to which degree each of these methods also activate keratinocytes, which importantly interact with Langerhans cells. It may be speculated that the different outcomes of the above methods, e.g., the relative inefficacy of the Viaskin® chamber, may well be explained by the assumption that hydration alone does not activate keratinocytes as much as tape stripping or abrasion. In fact, a heavily disrupted skin barrier has been observed to polarize the immune response toward Th1, whereas slight skin barrier disruption rather induces a non-inflammatory Th2/Treg–dominated response [23]. The evidence in this area is conflicting, as Mondoulet et al., in their mouse models of peanut allergy, find intact skin and un-stripped skin to be crucial for efficacy and safety of immunotherapy [65]. In contrast, our mouse models using OVA find no therapeutic effect if the skin was not tape stripped before allergen application [22].

Adjuvants in EPIT represent another strategy to enhance efficacy. Alum is the adjuvant used in the majority of marketed vaccines today [66]; however, it is not suitable for epicutaneous administration [67]. Thus far, cholera toxin (CT) and heat-labile enterotoxin (LT) have been successfully used as adjuvants in epicutaneous vaccination against infectious diseases of mice and humans [27••, 67, 68]. On the other hand, imidazoquinolines (TLR7 or -8 ligands) and CpG (TLR9 ligands) are currently being tested as adjuvants for epicutaneous vaccination against cancer [33, 69]. Our group recently tested the immune-enhancing and immune-modulatory potential of diphenylcyclopropenone when used as adjuvant in EPIT [22].

## Conclusion

In light of the increasing prevalence of allergic disease [70, 71], which strongly contrasts with the low percentage of patients choosing SCIT [4, 7], research during the next century should aim at optimization of current AIT methods in order to increase its attractiveness. Optimization of allergen immunotherapy should (1) deliver the allergen via a route that efficiently targets professional APCs, (2) use optimal adjuvants, (3) avoid allergen delivery to highly vascularized sites as to minimize systemic allergic side effects, and (4) be convenient for the patient, i.e., self-administrable and painless. Epicutaneous allergy immunotherapy holds promise in all four aspects: (1) the epidermis contains a high number of potent APCs, (2) adjuvants can be topically administered and/or physical or chemical trauma to keratinocytes may already act as an “physical adjuvant”, (3) the epidermis is non-vascularized, and (4) epicutaneous administration can be done at home and is painless.

Being afraid of needles, children would especially benefit most from a needle-free form of AIT [72], as AIT early in the course of allergic diseases has the potential to stop disease progression to asthma, which represents a considerable health burden. Such reasoning might highlight the development of a preventive, needle-free, patch-based allergy vaccine, accepted as a part of the WHO recommended “early childhood” vaccination program, to conquer the epidemic of the twenty-first century.

## Abbreviations

AIT: Allergen-specific immunotherapy
APC: Antigen presenting cell
CpG: Cytosin-phosphorus-CT cholera toxin
DC: Dendritic cell
EDS: Epidermal delivery system
EPIT: Epicutaneous allergy immunotherapy
IL: Interleukin
ILIT: intralymphatic allergen immunotherapy
i.m.: Intramuscular
LC: Langerhans cell
LT: Heat-labile enterotoxin
SALT: Skin associated lymphoid tissue
SCIT: Subcutaneous allergen immunotherapy
s.c.: Subcutaneous
SLIT: Sublingual allergen immunotherapy
TCI: Transcutaneous allergen immunotherapy
Th: Helper T cell
Treg: Regulatory T cell
